# Thromboembolism Early After Glucocorticoid Administration in Patients with Autoimmune Hemolytic Anemia

**DOI:** 10.1007/s44228-023-00043-9

**Published:** 2023-04-24

**Authors:** Kohei Shiroshita, Mikio Okayama, Hiroki Soma, Yuki Sato, Hiroyoshi Hayashi, Yuka Shiozawa, Shinichiro Okamoto, Ken Sadahira

**Affiliations:** 1 Division of Hematology, Kawasaki Municipal Kawasaki Hospital, 12-1 Shinkawa-dori, Kawasaki-city, Kanagawa 210-0013 Japan; 2 Division of General Internal Medicine, Kawasaki Municipal Kawasaki Hospital, 12-1 Shinkawa-dori, Kawasaki-city, Kanagawa 210-0013 Japan

**Keywords:** Autoimmune hemolytic anemia, Pulmonary embolism, Deep venous thrombosis, Glucocorticoid

## Abstract

Pulmonary embolism and deep venous thrombosis (PE/DVT) are well-known lethal complications in autoimmune hemolytic anemia (AIHA). However, the impact of their treatment is unclear. Here, we describe three elderly Japanese patients with AIHA who developed PE/DVT early after glucocorticoid administration. All patients presented with active hemolysis and high D-dimer levels upon admission. Thromboembolism was confirmed within 2 weeks after starting glucocorticoid, suggesting that both active hemolysis and glucocorticoid administration synergistically contributed to the development of PE/DVT. Clinicians should consider that such synergism may increase the risk of thromboembolism in patients with AIHA, and prophylactic anticoagulation is worth considering in patients after starting glucocorticoid.

## Introduction

Autoimmune hemolytic anemia (AIHA) is a rare hematological disease [1, 2]. Most AIHA cases are of warm-type, caused by an immunoglobulin G (IgG) autoantibody against red blood cell (RBC) surface antigens. RBC-binding autoantibodies are processed mainly by macrophages and neutrophils in the spleen [3], where extracellular hemolysis occurs. In addition, intravascular hemolysis by complement activation can also occur in about half of the patients with AIHA with high titers of IgG1 and IgG3 subclass autoantibodies that can activate the complement system [2, 4].

AIHA has been reported to increase the risk of thromboembolism [5], and recent literature supports this association [6–14]. Several mechanisms for thromboembolism development in patients with AIHA have been proposed. Those include phosphatidylserine exposure, free hemoglobin and heme release, RBC-derived microvesicles, and erythrocyte ADP and erythrocyte arginase. These factors can also synergistically induce thrombosis [15, 16]. Pulmonary embolism (PE) and deep venous thrombosis (DVT) also frequently occur in patients with AIHA [6–11, 17]. Although PE has been reported as the most common cause of death in these patients [18, 19] and has a lethal clinical course [20, 21], limited numbers of Japanese patients with such clinical courses have been reported in recent literature [12]. In addition, systemic glucocorticoid administration is the first-line therapy of AIHA, but it has been reported to associate with thrombosis [22, 23].

Herein, we describe three patients with newly diagnosed AIHA who developed thromboembolism early after starting systemic glucocorticoid.

## Case Presentations

Patient characteristics are summarized in Table 1.

**Table 1:** Patient characteristics in the three cases

Case No	Age	Gender	Hb [g/dL]	LDH [U/L]	T-bil/I-Bil [mg/dL]	D-dimer [μg/mL]	DAT	Presenting symptoms of thrombosis	Onset	Diagnosis of thrombosis	Therapy for thrombosis	Outcome
1	88	F	6.7	664	3.2/2.4	6.1	2 +	Right leg edema	On admission (excerbation at Day 9)	DVT	No treatment	Survived
2	77	F	5.2	962	8.2/7.3	14.3	3 +	Right leg edema	Day9	DVT	Apixaban	Survived
3	60	F	5.8	839	4.7/3.5	17.5	negative	Bilateral lower leg pain	Day6	DVT + PE	Apixaban	Survived

*Hb* hemoglobin, *T-Bil* total bilirubin, *I-Bil* indirect bilirubin, *DAT* direct antiglobulin test, *DVT* deep venous thrombosis, *PE* pulmonary embolism

### Case 1

An 88-year-old Japanese female was admitted to our hospital complaining of dyspnea for one week. Physical examination revealed mild edema in the right lower leg. The results of blood tests were remarkable for severe anemia (hemoglobin 6.7 g/dL; normal range 11.3–15.2 g/dL). Elevation of reticulocytes (4.7 × 10^5^/μL), indirect bilirubin (2.4 mg/dL; normal range, 0.2–1.0 mg/dL), lactate dehydrogenase (LDH) (644 IU/L; normal range, 124–222 IU/L), and D-dimer (6.1 μg/mL; normal range 0–0.9 μg/mL) were also noted. Haptoglobin and CD55^−^CD59^−^ cells were not detected. The COVID-19 PCR result was negative. DAT results were positive, and cold agglutinin disease (CAD) titer was within the normal range. Based on these findings, she was diagnosed with Coombs-positive AIHA. She was placed on prednisolone (PSL) 1 mg/kg/day, after which the hemoglobin level started rising. On the eighth day after starting PSL, she complained of progressive edema in the right lower leg. D-dimer elevated to 6.1 μg/mL, and ultrasound sonography revealed right calf and left popliteal venous thrombosis. The patient was diagnosed with DVT associated with AIHA. She was observed without anticoagulation, and the D-dimer level returned to within the normal range.

### Case 2

A 77-year-old Japanese female was admitted to our hospital complaining of a headache. The findings of the physical examination were unremarkable. The blood tests showed severe anemia (hemoglobin, 5.2 g/dL), elevation of reticulocytes (7.13 × 10^5^/μL), indirect bilirubin (7.3 mg/dL), LDH (962 IU/L), and D-dimer (14.3 μg/mL). Haptoglobin and CD55^−^CD59^−^ RBC cells were not detected. The COVID-19 PCR result was negative. DAT results were positive, but the CAD titer remained within the normal range. Based on the findings, she was diagnosed with Coombs-positive AIHA and was administered PSL 0.5 mg/kg/day. On day nine, after starting PSL, the patient developed right lower leg edema. The CL-β2GP1 antibody test results were negative. Lupus anticoagulant (LA) was positive but activated partial thromboplastin time was within the normal range. Ultrasonography revealed venous thrombosis in the right femoral and calf veins. Contrast-enhanced CT (ceCT) also revealed thrombosis from the right deep femoral vein to the right popliteal vein, but PE was not detected by ceCT. She was diagnosed with DVT associated with AIHA and was placed on apixaban. The right leg edema improved, and the D-dimer level returned to within the normal range.

### Case 3

**Fig. 1:**
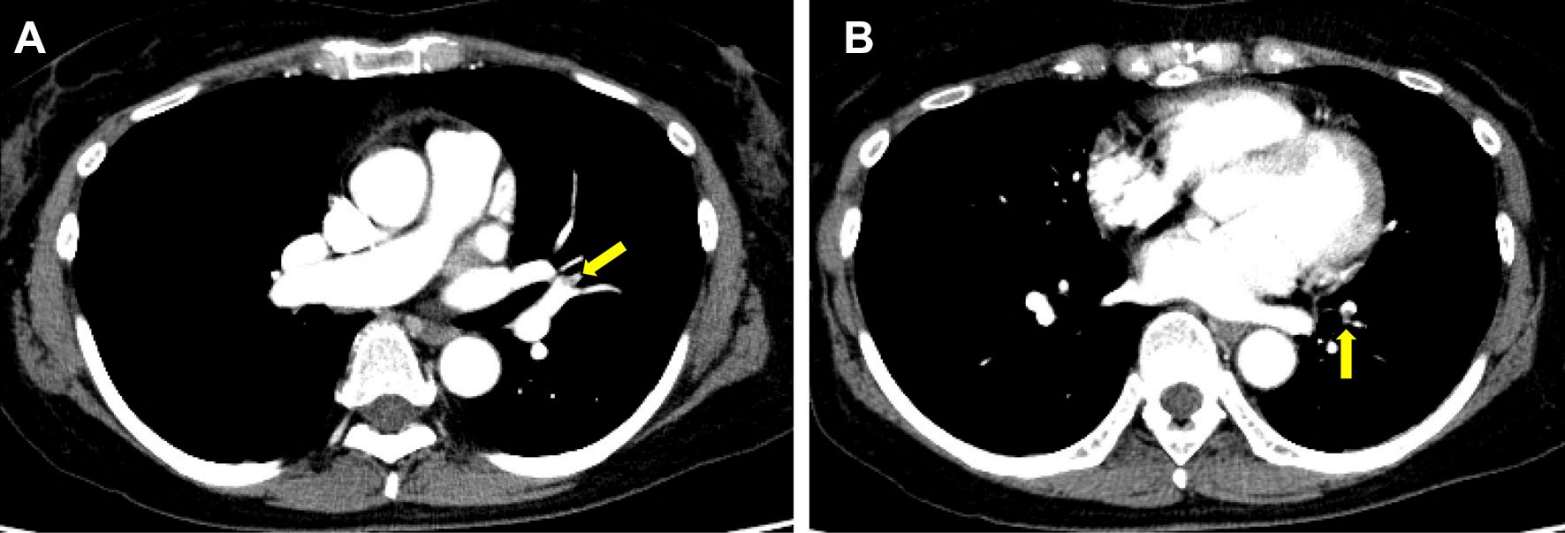
Pulmonary embolism in Case 3. **A**, **B** Contrast-enhanced computerized tomography revealed thrombosis in the left peripheral pulmonary artery (yellow arrow)

A 60-year-old Japanese female was admitted to our hospital complaining of dyspnea and dark urine. The patient’s medical history was unremarkable. The physical examination findings were normal, except for a systolic heart murmur. Blood test results showed macrocytic anemia (hemoglobin 5.8 g/dL), elevation of reticulocytes (2.95 × 10^5^/μL), indirect bilirubin (3.5 mg/dL), LDH (839 IU/L), and D-dimer (17.5 μg/mL). Haptoglobin and CD55^−^CD59^−^ RBC cells were not detected. The COVID-19 PCR result was negative. By both tube methods-based and column agglutination methods, DAT was negative. The mean fluorescence intensity difference of RBC-bound IgG after washing RBCs with phosphate-buffered saline was significantly elevated (48.8; normal range 5.5–16.0) [24]. The CAD titer was within the normal range. Based on these findings, she was diagnosed with Coombs-negative AIHA and placed on methylprednisolone pulse therapy, followed by PSL 1 mg/kg. The hemoglobin level started rising shortly; however, she complained of bilateral lower leg pain 6 days after starting PSL. Ultrasonography revealed bilateral calf and left popliteal venous thrombosis. ceCT also revealed multiple peripheral PE in the left lung (Fig. 1A, B). The D-dimer level remained high (13.2 μg/mL). LA and CL-β2GP1 antibodies were not detected, and protein C and S levels were within the normal range. She was diagnosed with PE/DVT associated with AIHA and was placed on apixaban. Complete resolution of the PE/DVT was confirmed by ceCT six months after treatment.

## Discussion

These AIHA cases emphasize that glucocorticoid administration soon after the diagnosis may increase the risk of PE/DVT.

Recent retrospective studies in Europe reported the characteristics of thromboembolism in patients with AIHA. The frequency of thrombosis-associated AIHA in European countries has been reported to be 11–27% [6–11, 13, 17]. One retrospective analysis showed that the rate of thrombotic events might be lower in Japan than in other countries [25].

Active hemolysis markers such as higher LDH levels, lower hemoglobin levels, and splenectomy have been identified as risk factors for thrombosis [7, 9, 10]. As seen in Case 2, some patients with AIHA and thrombosis were reported to be positive for LA; however, its association with AIHA remains controversial [7, 10, 17]. Intravascular hemolysis has also been reported as a risk factor for thrombosis [7, 9, 10], but other studies have not supported this finding [13]. This discrepancy may be explained by the differences in the study design, sample size, and availability of patient information.

Systemic glucocorticoid administration [23], old age [26], obesity [27], hospitalization, and malignancy [28] are well-known risk factors for PE/DVT. Our patients were elderly, but they were not obese and did not have a history of cancer and PE/DVT. As for the clinical course, the patients from Case 2 and Case 3 presented with leg edema after hospitalization, but they were not bedridden, and PE/DVT was documented within 2 weeks after PSL initiation. This raised the possibility that systemic glucocorticoid administration could also contribute to thromboembolism together with active or uncontrolled hemolysis. One case–control study showed that the highest risk of PE was at 30 days after starting glucocorticoid [22]. Another study also reported that the first 7 days after glucocorticoid administration increased the risk of PE/DVT among patients with malignancy [29]. These studies suggested that PSL drastically increases the risk for PE/DVT. A study focusing on patients with AIHA revealed that four out of 11 patients with AIHA were diagnosed with PE/DVT within 1 month, and other cases were diagnosed after 1 month [25]. In that study, almost all patients (98%) received PSL administration as the first-line therapy, and 10 of 11 presented active hemolysis at the diagnosis of PE/DVT. On another report, 7 out of 20 thrombotic events occurred at the time of AIHA diagnosis, and the median time to these events was 17.5 months [13]. Fattizzo et al. reported that 16 of 33 episodes (48%) of thrombotic complications occurred within the first 3 months. All studies suggested that clinicians should pay attention to the possibility of PE/DVT development not only at the time of diagnosing AIHA but also after starting its treatment. Although it was difficult to determine whether hemolysis or PSL definitively contributed to thrombosis in the acute phase, we speculate that the hypercoagulability due to active hemolysis itself can induce thrombosis, and PSL administration for AIHA treatment may synergistically promote thrombosis. Supporting this hypothesis, our cases presented a hypercoagulative state shown by a high D-dimer level on admission, but symptomatic PE/DVT occurred after starting PSL. However, it remains unclear why a limited number of patients with AIHA develop PE/DVT after glucocorticoid administration. Since the starting dose of PSL was decided by each physician’s clinical judgment, a higher PSL dose may affect the development of thrombosis in patients with AIHA. This point should be considered in future studies.

Although cases of splenic venous thrombosis, cerebral infarction, and acute coronary syndrome have also been reported [6–11, 17], most thromboembolisms in patients with AIHA are PE/DVT. Regarding PE, it has been reported to affect mainly pulmonary distal vessels like on patient 3 [20, 21, 30]. Concurrent PE might exacerbate clinical outcomes in patients with AIHA with severe right ventricular dysfunction, such as sudden death [19], hemodynamic compression [20], and cardiac arrest [21]. In addition, as distal PE may be easily missed [21], an imaging study should be performed.

In addition, the predictive scoring models of DVT, such as the Wells [31] and the Padua prediction scores [32], could also be applied. However, a previous study pointed out that the Padua score might not be able to predict PE/DVT in patients with AIHA [6]. Recently, an IMPROVE-DD score [33] of over 2.5 points has been reported to predict DVT in acutely ill patients within 48 h from admission [34]. In our cases, this score was three points (elderly and a D-dimer level twice as high as the upper normal limit). Based on this, we should have recognized that high D-dimer level is one of the important clues to suspect thrombosis in patients with AIHA. When clinicians detect high D-dimer levels in patients with AIHA on admission and early hospitalization, it is strongly recommended to perform ultrasonography of the lower legs to exclude DVT.

Whether the prophylaxis of thromboembolism should be given to all patients with AIHA remains to be elucidated [1, 2, 18], but several studies have reported the efficacy of prophylaxis for thrombosis. Hendrick reported that thrombotic events were lower in the prophylaxis than in the non-prophylactic group [11]. Fattizzo et al. also reported the efficacy of prophylaxis [7]. Other studies recommended anticoagulant therapy for active or severe hemolysis [6, 9, 15]. A prospective phase II study is currently in progress (NCT 05,089,227), and may clarify the efficacy, safety, and optimal indication for thromboprophylaxis.

In conclusion, we reported three AIHA patients complicated with PE/DVT after PSL administration. Although the number of cases is limited, our observations suggest that clinicians should always be aware of thromboembolism associated with AIHA during active hemolysis, especially after starting PSL, and keep higher suspicion of PE/DVT development in AIHA patients. A prospective cohort study is warranted to determine the precise incidence, risk factors, and pathophysiological mechanism of thrombosis in AIHA.

## Data Availability

Our manuscript has no associated data and materials.
